# Lung Ultrasound in Mechanical Ventilation: A Purposive Review

**DOI:** 10.3390/diagnostics15070870

**Published:** 2025-03-28

**Authors:** Amedeo Bianchini, Lorenzo Pintus, Giovanni Vitale, Elena Mazzotta, Cristina Felicani, Elena Zangheri, Maria Elena Latrofa, Cecilia Modolon, Rossella Pisano, Antonio Siniscalchi

**Affiliations:** 1 Post-Surgical and Transplant Intensive Care Unit, Department of Digestive, Hepatic and Endocrine-Metabolic Diseases, IRCCS Azienda Ospedaliero-Universitaria di Bologna, Via Giuseppe Massarenti, 9, 40138 Bologna, Italy; amedeo.bianchini@aosp.bo.ti (A.B.); rossella.pisano@aosp.bo.it (R.P.); antonio.siniscalchi@aosp.bo.it (A.S.); 2 Department of Medical-Surgical Specialties, Radiological Sciences, and Public Health, University of Brescia, 25121 Brescia, Italy; l.pintus001@studenti.unibs.it; 3 Internal Medicine Unit for the Treatment of Severe Organ Failure, IRCCS Azienda Ospedaliero-Universitaria di Bologna, 40138 Bologna, Italy; 4 IBD Unit, IRCCS Azienda Ospedaliero-Universitaria di Bologna, 40138 Bologna, Italy; elena.mazzotta@aosp.bo.it; 5 UOC Medicina ad Indirizzo Metabolico Nutrizionale, Policlinico di Modena, AOU Modena, Via del Pozzo, 71, 41125 Modena, Italy; cristinafelicani@gmail.com; 6 Anesthesiology and Pain Therapy, Sant’Orsola-Malpighi Hospital, University of Bologna, Via Giuseppe Massarenti, 9, 40126 Bologna, Italy; elena.zangheri@aosp.bo.it; 7 Pediatric Intensive Unit, IRCCS Azienda Ospedaliero-Universitaria di Bologna, 40138 Bologna, Italy; mariaelena.latrofa@aosp.bo.it; 8 Department of Radiology, IRCCS Azienda Ospedaliero-Universitaria di Bologna, Via Albertoni 15, 40138 Bologna, Italy; cecilia.modolon@aosp.bo.it

**Keywords:** lung ultrasound, mechanical ventilation, intubation, weaning, tracheotomy, diaphragm, ventilator-induced lung injury (VILI)

## Abstract

**Background/Objectives**: Lung ultrasound (LUS) has emerged as a crucial bedside tool for evaluating and managing patients with respiratory failure, particularly those receiving mechanical ventilation (MV). Its ability to rapidly characterise lung pathology, including extent, severity, and progression, has established LUS as a key diagnostic and monitoring modality in both hospital and home-care settings. **Methods**: This narrative review analyses the specific applications of LUS in the assessment and management of patients undergoing MV, aiming to optimise ventilatory strategies. **Results**: We examine the role of LUS in (1) identifying patients requiring MV; (2) guiding ventilator settings (Positive End Expiratory Pressure selection, inspiratory pressure adjustment, and patient-ventilator synchrony optimisation); (3) performing and monitoring recruitment manoeuvres; (4) assessing parenchymal damage and evaluating the response to medical and ventilatory therapies; (5) detecting ventilation-associated complications; (6) facilitating weaning from MV; and (7) assisting with airway management procedures, specifically tracheostomy. The utility of Transesophageal Lung Ultrasound (TELU) is also briefly discussed. **Conclusions**: This review highlights the potential of LUS to improve clinical decision making and patient outcomes in the context of MV.

## 1. Introduction

In recent years, lung ultrasonography (LUS) has become a landmark examination for evaluating respiratory failure patients [1]. Using ultrasonography, we can rapidly characterise lung damage and its extent, understand its severity, and follow its evolution directly at the patient’s bedside. These features are the reason for the widespread use of ultrasonography, which is currently one of the first tools used to manage patients with respiratory failure, both hospitalised and at home. Many authors have described the role of ultrasonography in the settings of different lung diseases to support the physician in the diagnosis, monitoring, and treatment of respiratory failure [1,2,3].

**Figure 1:**
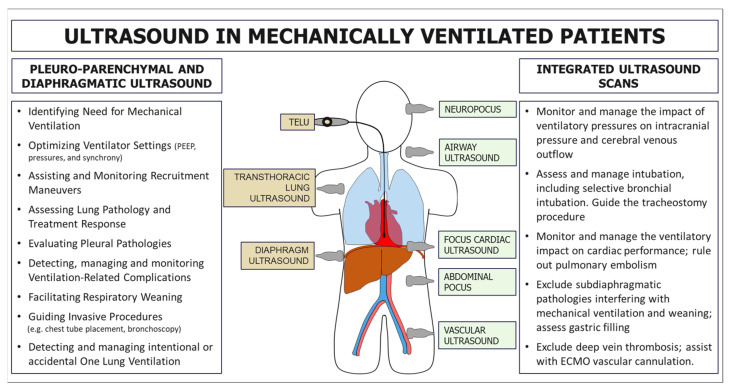
Primary applications of ultrasound in patients with mechanical ventilation. On the left, applications are related to the direct study of the pleuropulmonary and diaphragmatic regions using transcutaneous or transoesophageal scans. On the right, some potential ultrasound applications related to the study of other systems and organs are useful for an integrated assessment and management of patients with mechanical ventilation.

Mechanical ventilation (MV) is a fundamental intervention in managing patients with respiratory failure, and its careful optimisation is crucial for improving clinical outcomes. LUS has gained recognition as a powerful adjunct for supporting clinicians in various aspects of MV (Figure 1). Despite its significant potential, the current literature presents a fragmented analysis of its applications, limiting its systematic adoption in routine clinical practice. In this concise review, we delineate the specific role of LUS in evaluating and managing mechanically ventilated patients, aiming to provide a structured framework to enhance ventilatory strategies and clinical decision making.

We have considered various aspects of ultrasound’s role in MV, including its utility in identifying patients requiring ventilatory support and adjusting ventilator parameters, such as Positive End Expiratory Pressure selection (PEEP) selection, Inspiratory Pressure (InsP), and optimising ventilator–patient synchrony. Furthermore, the use of ultrasound in recruitment manoeuvres, such as achieving opening pressure and pronation, as well as in the progressive monitoring of parenchymal damage and the assessment of the effectiveness of medical and ventilatory therapies, is explored. The study also examines the role of ultrasound in evaluating complications related to ventilation, the respiratory weaning process, and airway management, with particular attention to tracheotomy. Finally, the most relevant aspects of the Transesophageal Lung Ultrasound (TELU) approach are analysed.

## 2. Materials and Methods

**Study Design:** This is a purposive review analysing LUS applications in managing MV patients.

**Literature Search:** A search was conducted by 31 December 2024, including e-Pub published articles in PubMed, MEDLINE, and the Cochrane Library, using keywords such as “lung ultrasound”, “mechanical ventilation”, “weaning”, “Acute Respiratory Distress Syndrome”, “ARDS”, ”Positive End Expiratory Pressure”, “PEEP”, ”Transesophageal Lung Ultrasound”, “TELU”, “Ventilator-Induced Lung Injury”, “VILI”, and “diaphragm”.

**Study Selection:** Studies in the English language were included if they discussed the application of LUS in adult patients undergoing MV.

**Data Extraction and Synthesis:** Data extraction focused on patient population, clinical setting, specific LUS applications, and key findings related to the review’s objectives. Specifically, the synthesis covered the roles of LUS in the following:

1. Identifying patients needing MV.
2. Guiding ventilator settings (PEEP, InsP, and synchrony).
3. Monitoring recruitment manoeuvres.
4. Assessing parenchymal damage and therapy response.
5. Detecting ventilation-associated complications.
6. Facilitating respiratory weaning.
7. Assisting airway management (tracheostomy).
8. The utility of TELU.

Finally, data were synthesised narratively, identifying common themes and summarising the available evidence for each application of LUS in MV.

## 3. Results

### 3.1. Role of Ultrasound in Identifying Patients Needing Mechanical Ventilation

The ability to anticipate whether a patient will require MV is crucial for setting up appropriate monitoring, ensuring proper patient placement in the most suitable unit, and preparing for ventilatory support.

Several point-of-care ultrasound protocols have emerged in recent years to simplify the ultrasound approach for patients with respiratory failure. One of the first is the Blue Protocol [4]. The Blue Protocol demonstrates how ultrasound can quickly identify most pulmonary pathological conditions (90%).

In patients with acute respiratory failure, ultrasound diagnosis of pneumothorax or ultrasound signs of pulmonary embolism or asthma guide specific therapies or procedures over MV (e.g., chest drainage, thrombolysis, bronchodilators) [1,4,5]. Conversely, the ultrasound finding of signs compatible with pulmonary flogosis or acute pulmonary oedema prompts the physician to consider early invasive or non-invasive ventilatory support.

**Figure 2:**
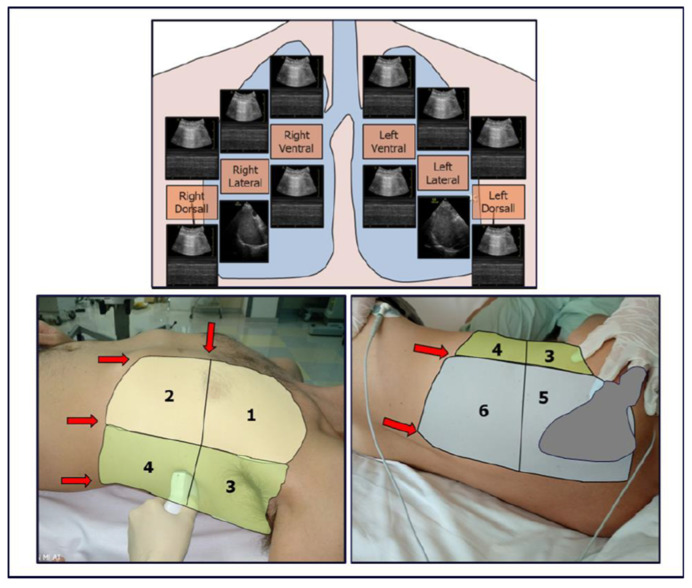
Thoracic zones (1–2 anterior, 3–4 lateral, 5–6 dorsal) are used to calculate the Classic Lung Ultrasound Score. A second operator is required to assist in assessing the dorsal zones in a supine patient undergoing mechanical ventilation. The presence of ribs and the scapula limits access to the pleural surface.

**Figure 3:**
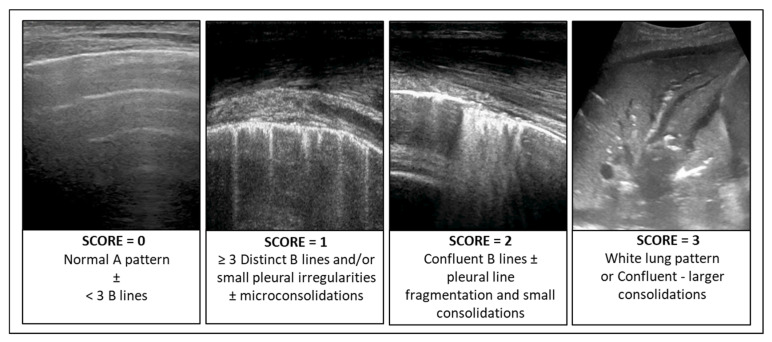
Ultrasound findings for calculating the Lung Ultrasound Score (LUSsc 0–36).

In patients with infectious pulmonary disease, the LUS score (LUSsc) allows for the characterisation of the extent of lung lesions and the severity of the condition directly at the patient’s bedside. The LUSsc is determined based on four ultrasound patterns of lung pathology assessed in 12 lung areas (Figure 2) [6]. The LUSsc enables the physician to quickly assess the degree of parenchymal compromise to choose the most appropriate monitoring and ventilatory support. Figure 3 explains ultrasound findings for calculating the LUSsc. A LUSsc ≥ 18 on a scale from 0 to 36 correlates with the need for intubation [7]. Mongodi et al.’s more updated version of the LUSsc distinguishes lung aeration loss based on the percentage of pleura affected by artefacts, such as B-lines and subpleural consolidations [8]. This approach provides a more remarkable inter-operator agreement.

The extent of lung damage in an area ≥ two-thirds of the explorable parenchyma can predict the need for tracheal intubation [9].

Using the Visual Lung Ultrasound Protocol (VLUP), the extent of lung pathology can be assessed through a point-of-care visual approach, allowing for a quicker understanding of whether the patient will require intubation (LUSq ≥ 2/3) [10].

Furthermore, the failure to reduce the LUSsc after recruitment manoeuvres, such as pronation or non-invasive ventilation, can predict the need for intubation and invasive mechanical ventilation [11].

### 3.2. Role of Ultrasound in Setting Ventilator Parameters: Choosing PEEP, Insufflation Pressures, and Optimizing Ventilator–Patient Synchrony

#### 3.2.1. Alveolar Recruitment and Choice of PEEP

The role of ultrasound in determining ventilatory pressures has gained significant attention during the recent COVID-19 pandemic. Early in the pandemic, the importance of LUS emerged in differentiating patients into two phenotypes [12]: those responsive to high PEEP [H phenotype] and those responsive to low PEEP [L phenotype]. In this context, ultrasound has been crucial in managing ventilation for patients with SARS-CoV-2 pneumonia.

As described by Costamagna et al., ultrasound allows rapid identification of the Acute Respiratory Distress Syndrome (ARDS) phenotype by distinguishing ARDS with focal involvement from ARDS with non-focal (or diffuse) involvement. A LUSsc in the anterior quadrants > 2 identifies a non-focal ARDS morphology (sensitivity = 0.95, specificity = 1.00) [13].

The focal ARDS pattern is more prone to overinflation with high PEEP; however, it responds well to the prone position. Conversely, the diffuse ARDS pattern is less prone to overinflation with high PEEP but does not respond to the prone position [14].

For this reason, some authors argue that “personalized mechanical ventilation” based on lung morphology (focal vs. non-focal) could improve survival outcomes in ARDS compared to the standard of care outcome. The report of the LIVE study by Constantin et al. shows that the mortality of patients with personalized ventilation based on lung phenotype is lower than that with the standard approach if “misclassified patients” are excluded [15,16].

**Figure 4:**
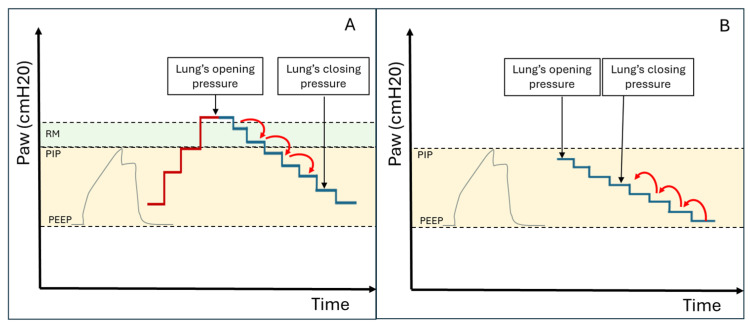
(**A**) Ultrasound-guided pulmonary recruitment according to the protocol by Tusman G et al. In this case, the opening pressure is reached with the Recruitment Manoeuvre (RM), and the closing pressure is subsequently identified by gradually reducing the airway pressure. (**B**) Ultrasound-guided recruitment protocol when InsP exceeds the lung opening pressure. In these cases, alveolar reopening occurs with each inspiratory effort, followed by consolidation during the expiratory phase (Video S1). The protocol involves gradually increasing Positive End Expiratory Pressure (PEEP) under ultrasound guidance until the lung closing pressure is surpassed. Ultrasound is then used to verify the maintenance of alveolar ventilation throughout all ventilatory phases (Videos S2 and S3). PIP = Peak Inspiratory Pressure.

**Figure 5:**
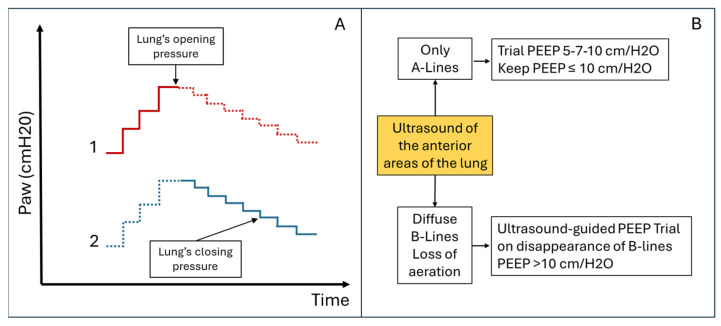
Comparative Alveolar Recruitment Protocols. (**A**) Ultrasound-guided pulmonary recruitment according to Tusman G et al. protocol [17]. Objective: Alveolar recruitment through ultrasound identification of lung opening and closing pressures. Area to be scanned: Consolidated areas. (**B**) Ultrasound-guided pulmonary recruitment according to the Belaïd Bouhemad et al. protocol [19]. Objective: Titrated alveolar recruitment focused on the anterior, less consolidated, lung areas to limit overdistension. Area to be scanned: anterior lung quadrants. This protocol involves recruitment trials with PEEP ≤ 10 cm/H_2_O if there are A-lines or focal areas of aeration loss in the anterior lung areas. PEEP > 10 cm/H_2_O (with increments of 3–4 cm/H_2_O) will be used if the anterior areas are consolidated.

MV protocols designed to identify the optimal end-expiratory pressure have been proposed. These protocols aim to recruit hypoventilated lung areas and subsequently maintain them aerated. The main ventilation protocols involve progressively increasing inspiratory pressures until ventilation resumes in consolidated lung areas (this pressure can be used for subsequent recruitment manoeuvres). Subsequently, the pressure is reduced again until lung consolidation reoccurs, allowing the identification of the airway closing pressure. The optimal PEEP value is 2 cmH20 above the airway closing pressure (see Figure 4A and Figure 5A) [17].

In patients where Peak Inspiratory Pressure (PIP) consistently exceeds the opening pressure of the lung (causing alveolar opening and closing with every breath—Video S1), PEEP can be gradually increased under ultrasound guidance until the lung closing pressure is surpassed (Figure 4B). Ultrasound is then used to verify the maintenance of alveolar ventilation throughout all ventilatory phases. Videos S2 and S3 demonstrate the application of this ultrasound-guided PEEP increment protocol in a patient on non-invasive ventilation (Video S2) and in a patient on invasive ventilation (Video S3). This protocol is also helpful in cases of persistent hemodynamic instability (limiting the Tusman et al. protocol) or when high respiratory pressures are contraindicated due to pulmonary pathologies (e.g., pneumothorax) or the presence of severe coexisting conditions in other organs (e.g., acute brain injury).

However, the alveolar recruitment protocols described above do not consider the risk of hyperinflation and ventilator-induced lung injury (VILI). Hyperinflation is common in patients with heterogeneous lungs subjected to MV at high pressures. Some authors describe hyperinflated areas as regions with an increased number of A-lines (>5) associated with reduced pleural sliding [18] (Video S4). However, other authors argue that LUS [19] does not adequately study hyperinflated areas. To limit the phenomenon of hyperinflation, ultrasound protocols for PEEP titration have been proposed, which focus on studying the anterior lung areas rather than the consolidated areas [6] (see Figure 5B). LUS also allows early identification of patients at higher risk of developing hyperinflation, such as patients with focal ARDS patterns or obstructive respiratory disease [5,20]. In these patients, LUS may be essential to monitor the onset of overinflated areas during mechanical ventilation.

The role of ultrasound in alveolar recruitment has also been studied during intraoperative MV. Some authors show how ultrasound-guided alveolar recruitment during surgery can improve alveolar aeration and reduce atelectasis at the end of the operation with lasting benefits in the postoperative period [21,22].

Alveolar recruitment achieved by increasing PEEP, as estimated through pressure-volume curves, corresponds to the evaluation obtained by LUSsc. LUSsc also correlates with the Recruitment-to-Inflation Ratio (R/I ratio) [23].

Before proceeding with alveolar recruitment manoeuvres by increasing airway pressure, it is recommended that the airways be scanned to verify patency by looking for aerated bronchograms (Video S5A) and/or excluding fluid bronchograms (Video S5B). In cases of fluid bronchograms (bronchial obstruction), bronchial clearance manoeuvres, such as bronchoscopy, may be beneficial before proceeding with recruitment. Furthermore, it may be helpful to assess ventilation-induced changes in organ blood flow and perfusion before, during, and after increasing ventilatory pressures, aiming to minimise associated detrimental effects. For instance, focused echocardiography (FOCUS), can be employed to evaluate the influence of ventilatory pressures on right ventricular outflow in patients with right ventricular dysfunction [24].

In this context, integrating thoracic ultrasound with echocardiographic scans can aid in selecting the most suitable level of PEEP to recruit lung parenchyma while maintaining good right ventricular function (e.g., in patients with ARDS and right ventricular dysfunction related to pulmonary hypertension or severe IT). Other examples of combining cardiac FOCUS with thoracic ultrasound include choosing ventilatory pressures in patients with a patent foramen ovale to limit right-to-left shunting, optimising PEEP in patients with left ventricular heart failure to alleviate pulmonary congestion, and ruling out severe hypovolaemia before performing alveolar recruitment manoeuvres in critically ill patients to lessen the impact of ventilation on ventricular filling.

Integrating a Neuro-Point-of-Care Ultrasound (neuro-POCUS) assessment with thoracic ultrasound may also play a role in determining ventilatory pressures in critically ill patients. Critically ill patients often require prolonged protective ventilation, using high PEEP and low tidal volume to reduce ventilator-induced lung injury. However, these techniques and the prone position can impair cerebral venous drainage and elevate intracranial pressure (ICP). Additionally, hypercapnia resulting from low tidal volumes may further increase cerebral blood flow, exacerbating ICP elevation. This phenomenon is particularly detrimental in patients with acute brain injury. A neuro-POCUS examination integrated with thoracic ultrasound can be valuable in optimising ventilatory pressures to mitigate ICP elevation. By analysing the Doppler spectrum of cerebral arteries (e.g., the Middle Cerebral Artery), assessing cerebral venous outflow (e.g., via transcranial Doppler of the vein of Rosenthal or ultrasound evaluation of the internal jugular veins), and measuring the optic nerve sheath diameter, it is possible to monitor the impact of mechanical ventilation on cerebral venous drainage and ICP. This integrative approach may aid in optimising ventilatory settings and guiding alveolar recruitment manoeuvres [25] (Figure 1).

Pronation position is another technique for alveolar recruitment. Consolidated lung regions in dependent areas can be reventilated by pronating the patient, improving respiratory gas exchange. However, pronation manoeuvres in mechanically ventilated patients are complex and potentially risky. LUS helps recognise patients who are responsive to pronation (focal pattern ARDS and posterior predominant consolidations) and for monitoring the effects on alveolar reventilation [22]. LUSsc variation is associated with pronation responsiveness; prone-induced lung inflation greater than 500 mL can be accurately estimated by an LUS reventilation score of 10 or greater [26,27].

#### 3.2.2. Choice of Inspiratory Pressure

In addition to identifying the optimal PEEP, LUS allows for identifying the best InsP in patients receiving assisted ventilation. The study of diaphragmatic thickness and activity helps determine whether the patient receives adequate, excessive (with a risk of damaging diaphragmatic tropism), or insufficient (with a risk of muscle exhaustion) ventilatory support.

The Diaphragmatic Thickening Fraction (TFdi) is defined using the following formula:

TFdi = [(End-inspiratory diaphragm thickness − End-expiratory diaphragm thickness/

End-expiratory diaphragm thickness)] × 100%

A Diaphragmatic Thickening Fraction (TFdi) < 15% is associated with over-assistance or diaphragmatic dysfunction, while TFdi > 30–50% indicates insufficient InsP [28].

As described by Tuinman et al. [29], TFdi is a parameter whose range varies depending on several factors, including patient positioning, respiratory effort, and diaphragmatic dysfunction. In intensive care unit patients in a semirecumbent position, a TFdi > 30% is considered normal. In mechanically ventilated patients, a TFdi < 15% is associated with over-assistance or diaphragmatic dysfunction (a dysfunctional diaphragm exhibits TFdi < 15% both during mechanical ventilation and spontaneous breathing trials; in contrast, a normally functioning diaphragm shows TFdi < 15% during mechanical ventilation with excessive pressure support but thickens appropriately during spontaneous breathing trials). A TFdi value > 30–50% indicates increased diaphragmatic activation by the patient due to insufficient inspiratory pressure (TFdi values decrease after increasing inspiratory pressure, reflecting improved ventilatory support). Ultrasound assessment of intercostal muscle activity (accessory respiratory muscles) can also help determine the optimal inspiratory pressure. Since intercostal muscles typically contribute minimally to the ventilatory dynamics, their activation is commonly observed in cases of insufficient inspiratory pressure (see Video S6).

In the same way, the Intercostal Thickening Fraction (TFic) is defined as a measurement of the change in thickness of the parasternal intercostal muscle between inhalation and exhalation. It is calculated as follows:

TFic = [(End-inspiratory muscle thickness − End-expiratory intercostal muscle thickness/

End-expiratory intercostal muscle thickness)] × 100%

TFic typically ranges from 0 to 4%, whereas TFic > 8% correlates with inadequate ventilatory support [30].

Ultrasound assessment of the activity of the intercostal muscles (accessory muscles) can also help determine the best InsP. Activation of the intercostal muscles is common in cases of insufficient InsP (see Video S6). The Intercostal Thickening Fraction (TFic) ranges typically from 0 to 4%, while TFic > 8% correlates with insufficient support [30].

#### 3.2.3. Ventilator–Patient Synchrony

The study of respiratory muscles can also help evaluate synchrony between the patient and the ventilator. Asynchrony is common (about 50% of patients on MV) and is associated with a worse clinical outcome. An integrated study of diaphragmatic activity and ventilatory mechanics can document asynchrony and guide modifications to ventilator settings [30].

### 3.3. Role of Ultrasound in Monitoring Parenchymal Damage and Ventilatory/Medical Therapy

Over the years, thoracic ultrasound has proven invaluable for bedside monitoring of thoracic pathologies. Various studies have shown its utility in tracking the progression of pulmonary conditions like bronchopneumonia, cardiogenic pulmonary oedema, extravascular lung water, pneumothorax, and pleural effusion extension [31,32].

The evolution of LUS findings correlates with lung involvement as seen on computed tomography (CT) and X-rays. Monitoring pleuroparenchymal pathology helps guide ventilatory, pharmacological, and surgical interventions [33,34].

**Figure 6:**
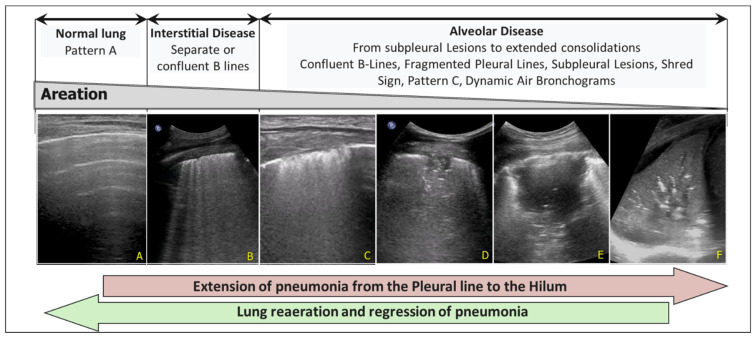
Progression of pneumonia from the pleural line to the pulmonary hilum. (**A**) Pattern A (Normal). (**B**) Interstitial Disease with separate or confluent B-lines. (**C**) Initial alveolar involvement. Confluent B-lines, Fragmented Pleural Lines and Subpleural Lesions. (**D**,**E**) Extension of pneumonia: subpleural hypoechoic lesion, Shred Sign, hyperechoic spots, confluent B lines. (**F**) Extended consolidation and dynamic air bronchograms.

For example, the increase in pulmonary consolidation in successive evaluations in a patient with prolonged mechanical ventilation (Figure 6) guides the physician in adjusting PEEP, performing alveolar recruitment manoeuvres (see above), or revising the prescribed antibiotic therapy.

On the other hand, the increase in B-lines in a patient with pulmonary oedema suggests increasing PEEP, diuretic therapy or cardiovascular support. The expansion of a pleural effusion suggests thoracentesis and adjustments to diuretic therapy. In contrast, the progression of a pneumothorax (through serial assessment of the lung point displacement) in a mechanically ventilated patient suggests the placement of a chest drain, surgical evaluation and modification of ventilation pressures. Finally, some authors have proposed the daily LUSsc to monitor global lung recovery in ARDS patients on extracorporeal membrane oxygenation (difficulty in performing CT scans) [35,36].

### 3.4. The Role of Ultrasound in Ruling out Complications from Mechanical Ventilation

Complications secondary to MV are various and affect the lungs and other organs. The most common complications include overinflation, barotrauma, volutrauma, atelectotrauma, ventilator-associated pneumonia, diaphragmatic atrophy, hypoventilation (e.g., due to selective intubation or low airway pressures), pneumothorax, and subcutaneous emphysema. Ultrasound can evaluate and prevent most of these complications [32,37,38].

The utility of LUS in diagnosing and monitoring pneumothorax (absence of pleural sliding, lung pulse, and B-lines, with the presence of a lung point) and subcutaneous emphysema caused by parenchymal trauma from volume or pressure in mechanically ventilated patients is well established [1,4,37].

As mentioned earlier, LUS is also beneficial for PEEP titration to avoid alveolar opening and closing cycles, thereby preventing atelectotrauma (Videos S2 and S3). This repeated trauma, and the resultant localised inflammatory response are key mechanisms underlying VILI.

In mechanically ventilated patients, ultrasound aids in identifying hyperinflated areas (Video S4), titrating ventilatory pressures to reduce hyperinflation and early detection of patients with obstructive lung diseases at risk for air trapping (as previously discussed).

LUS also facilitates titration of support pressures to avoid over-assistance, which causes diaphragmatic atrophy (Video S7) or excessive respiratory effort, leading to muscular exhaustion (maintaining TFdi values between 15% and 30%) [30].

**Figure 7:**
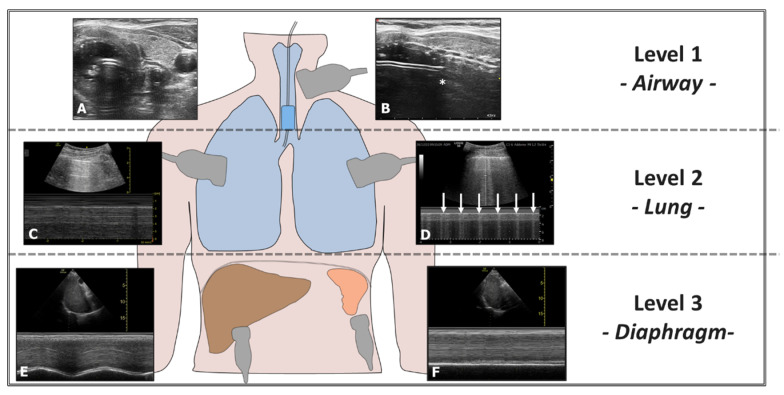
Evaluation of Right-Sided Selective Intubation with Accidental or Intentional Exclusion of the Left Lung. Evaluation performed at three levels: 1st Level—Airways: (**A**) Transverse scan of the airways showing the ETT in the trachea (Bullet Sign). (**B**) The position of the upper edge of the ETT cuff (*) is located too caudally relative to the cricoid. 2nd Level—Lung: (**C**) Presence of the Sea Shore Sign in the ventilated lung. (**D**) Lung Pulse observed in the excluded lung (arrows). 3rd Level—Diaphragm: (**E**) Diaphragm with regular excursions in the ventilated lung. (**F**) Immobile diaphragm in the excluded lung.

Ultrasound can also confirm proper positioning of the endotracheal tube (ETT) without radiological imaging and quickly exclude accidental bronchial intubation. Accidental selective intubation causes atelectasis and respiratory insufficiency and promotes lung infection. Ultrasound can exclude this condition through a three-level assessment: airway, lung, and diaphragm (Figure 7). Airway ultrasound is a technique increasingly used in recent years. The American Heart Association (AHA) recommends the ultrasound assessment of successful orotracheal intubation when capnometry is unavailable [39]. Ultrasound can also assist in repositioning the ETT accurately using an ultrasound-guided technique (Video S8) [40].

Ventilator-associated pneumonia has several causes beyond pulmonary exclusion, including microaspiration. Ultrasound allows for assessing gastric filling and its ultrasound-guided drainage (up to complete gastric emptying), which is highly useful in reducing the risk of aspiration (Video S9). Additionally, airway ultrasound can evaluate the accumulation of secretions above the ETT cuff, enabling appropriate clearance measures [41].

Mongodi et al. suggest the role of LUS monitoring in the early diagnosis of ventilator-associated pneumonia. In the intensive care population, an increase in the LUS score compared to baseline should raise suspicion of superinfection and lead to the search for specific ultrasound signs suggestive of pneumonia (i.e., presence of consolidation and dynamic linear-arborescent air bronchogram) [32].

LUS is also helpful in evaluating complications involving extrapulmonary organs and systems, such as functional right ventricular failure, the presence of intracardiac shunts, splanchnic venous congestion (VeXus Score), and increased intracranial pressure [42,43].

### 3.5. Role of Ultrasound in Respiratory Weaning

Respiratory weaning often presents a significant challenge for physicians, involving multiple interacting systems; respiratory, cardiovascular, neuromuscular, and abdominal. POCUS ultrasound can assist in the weaning process from MV by evaluating these systems individually. To this end, Tuinman et al. proposed an ultrasound-based ABCDE approach for respiratory weaning [30] as follows:

*A. Aeration Score—Lung Parenchyma and Pleural Effusion Evaluation:* A Lung Ultrasound Score (LUSsc) > 17, the appearance of more than six B-lines during the Spontaneous Breathing Test (SBT), and significant pleural effusion have been associated with extubation failure [7,44].

*B. Below the Diaphragm:* Ultrasound evaluation of the upper abdominal quadrants can identify ascites, masses, or intra-abdominal abscesses that may impact respiratory mechanics.

*C. Cardiac Function:* Assessment of the left ventricle’s systolic and diastolic function is critical, as both systolic and diastolic dysfunction can affect respiratory autonomy due to heart–lung interaction.

*D. Diaphragm:* Ultrasound evaluation of diaphragmatic thickness (indicating muscle trophism) and diaphragmatic thickening fraction and excursion (indicating functional capacity) are crucial parameters in respiratory weaning. Measurements are taken using a linear probe in the intercostal view (mid-axillary line). A significantly reduced thickness or a TFdi < 30–36% during SBT correlates with weaning failure. (Videos S6 and S7B). A diaphragmatic excursion is assessed with a low-frequency probe in a subcostal view, where a bilateral excursion < 10 mm is linked to failed weaning.

*E. Extra-diaphragmatic Muscles:* It is critical to evaluate accessory respiratory muscle activation during SBT. Hyperactivity of these muscles indicates reduced diaphragmatic activity and increased respiratory effort, which correlate with a higher likelihood of extubation failure (Video S6).

A systematic review and meta-analysis by Llamas-Álvarez et al., involving 1071 intubated Intensive Care Unit patients, showed that LUS combined with targeted diaphragm ultrasound could help predict weaning outcomes [45]. However, the limited number of studies and the variability in patient subpopulations necessitate cautious interpretation. The ultrasound parameters utilised in respiratory weaning are currently characterised by inconsistent cut-off values, mainly due to the lack of standardised protocols for their derivation [30]. This variability arises from assessments conducted under differing conditions, such as variations in patient positioning, levels of respiratory support, and the duration of spontaneous breathing trials (SBTs). Therefore, the operators must integrate ultrasound findings from the ABCDE protocol with clinical findings to guide ventilator weaning.

Ultrasound can also support respiratory weaning by facilitating tracheostomy, as demonstrated by protocols described in the literature for ultrasound-guided percutaneous tracheostomy [40,46,47]. Percutaneous tracheostomy (PDT) is commonly performed using anatomical landmarks, bronchoscopy, or ultrasound guidance. Although the overall complication rate is low, severe complications can occur, including airway loss, massive bleeding, pneumothorax, tracheal injuries, cannula displacement, or occlusion [47].

Ultrasound allows for the evaluation of the airways before performing PDT, identifying the precise location of the tracheal and laryngeal structures and highlighting pathological anatomical alterations (e.g., tracheal displacement and neck masses) or physiological variations (e.g., cranial position of the brachiocephalic trunk). Ultrasound can, thus, guide the execution of PDT and allows for the detection of both early and late complications (e.g., bleeding and tracheal stenosis) [40,47,48]. Ultrasound-guided percutaneous tracheostomy is safe and comparable to bronchoscopy-guided PDT in terms of major and minor procedural or clinical complications, and it reduces minor complications when compared to anatomical landmark-guided PDT [49]. Ultrasound-guided PDT offers several advantages over bronchoscopy, including greater availability in intensive care settings and reduced personnel requirements; however, this technique has not been standardised and involves a steep learning curve [48].

### 3.6. Transesophageal Lung Ultrasound (TELU)

The literature describes TELU as an alternative to transthoracic pleuro-parenchymal ultrasound in specific contexts [50].

TELU provides real-time information directly at the patient’s bedside in intensive care and the operating room. TELU facilitates cardiac and pulmonary evaluation while rapidly assessing heart–lung interaction during respiratory failure and can also help assess lung recruitment [6].

The transesophageal ultrasound probe (4 MHz) is positioned close to the posterior regions of the lung parenchyma, allowing better visualisation of pathological findings in this area (Video S5B and S10). The examination follows a systematic approach, like transthoracic lung ultrasound, dividing each lung along the craniocaudal axis into three regions: apical, middle, and basal. Key landmarks include the origin of the left subclavian artery for the apical region, the superior pulmonary veins for the middle region and the inferior vena cava-right atrial junction for the basal region.

Starting from these landmarks, a 90° electronic probe rotation allows longitudinal lung scanning. From the cardiocentric starting position (12:00), the probe is rotated counterclockwise to examine the left lung, continuing to the right lung, completing a full rotation. Since lung segments cannot be easily identified, the ultrasound beam’s position is described using the probe’s handle as a clock reference: Right lung, anterior at 2:00, lateral at 3:00, posterior at 4:00. Left lung, anterior at 10:00, lateral at 9:00, posterior at 8:00 [2].

TELU offers key advantages, including bedside accessibility without needing direct chest access and real-time feedback. The probe’s proximity to the posterior lungs enables better imaging of pleural effusions, consolidations and B-lines, particularly in posterosuperior zones that are typically blind spots for transthoracic ultrasonography [50].

However, TELU has significant limitations. It is more invasive, less validated, and lacks extensive supporting evidence. It is also less effective for detecting right-sided pathologies and cannot access the anterior and lateral lung regions. Like transthoracic ultrasonography, TELU relies heavily on artefact interpretation and is operator dependent.

## 4. Conclusions

Lung ultrasound has emerged as a pivotal modality in managing patients with respiratory failure, particularly those undergoing mechanical ventilation.

This review aims to explore the potential applications of LUS in ventilated patients, emphasising its role in guiding clinical decisions and optimising respiratory support. Specifically, LUS assists in determining the need for ventilatory intervention and setting ventilator parameters, such as positive end-expiratory and insufflation pressures. This approach helps mitigate ventilator-associated lung injury and enhances ventilator–patient synchrony. Furthermore, LUS proves invaluable in identifying and monitoring complications related to MV and assessing the weaning process from mechanical ventilation.

Despite the promising applications of thoracic ultrasound in mechanically ventilated patients, several factors may hinder its practical use in critically ill subjects. Common limiting factors include the limited availability of ultrasound machines, time constraints (as the procedure can be time-consuming), the need for contact isolation in cases of highly resistant infections, and restricted acoustic windows due to factors such as mandatory postures (e.g., prone positioning) or the presence of dressings, drains, and braces. Additionally, some scans, particularly in dependent areas, or ultrasound assessments during specific manoeuvres (e.g., increased ventilatory pressures), may require multiple operators. Other limitations include the subjectivity of data interpretation, difficulty quantifying results, and the need for accredited and dedicated training programs, as operator dependency and inter-individual variability can affect diagnostic accuracy. A recent survey identified several key barriers to the accreditation of a thoracic ultrasound training program, including a lack of courses (52%), a lack of mentors (93%), and difficulty arranging directly supervised scans (73%). However, despite these limitations, the increasing availability of high-performance ultrasound machines at lower costs, the integration of standardised protocols, the development of advanced image analysis software, and the growing focus on structured training programs may help overcome these challenges, enhancing reliability and facilitating broader adoption of the technique [51].

LUS applications rapidly expand, transcending traditional pulmonary assessments to encompass extra-pulmonary ultrasound techniques, thus facilitating a more comprehensive diagnostic evaluation. However, the effectiveness of these applications depends on their precise correlation with well-defined clinical questions. Adopting an integrated approach that combines LUS findings with other diagnostic modalities and thorough clinical evaluations is essential to achieve optimal patient outcomes.

## Data Availability

The original contributions presented in this study are included in the article/Supplementary Materials. Further inquiries can be directed at the corresponding author.

## Supplementary Materials

The following supporting information can be downloaded at: https://www.mdpi.com/article/10.3390/diagnostics15070870/s1, Video S1: Patient in a metabolic coma due to End-Stage Liver Disease (ESLD) receiving mechanical ventilation (PSV: Pins 12, PEEP 5). The peak inspiratory pressure (PIP) surpasses the lung opening pressure, leading to lung recruitment with each breath. Ultrasound scanning was conducted in the fourth thoracic quadrant (see Figure 2) using a right intercostal approach. The video depicts the basal region of the right lung collapsing at the end of each expiratory phase and reopening during inspiration. The repetitive opening and closing of the lung may induce atelectotrauma. Video S2: A patient experiencing respiratory failure after abdominal surgery. An ultrasound-guided increase in PEEP during Non-Invasive Ventilation (NIV) was carried out, resulting in complete pulmonary re-expansion across all ventilatory phases (as illustrated in the right panel). The three video panels display the basal region of the right lung. Ultrasound scans were carried out in the same fourth thoracic quadrant (see Figure 2) using a right intercostal approach at various time points. The left panel illustrates the lung base collapsing at the end of each expiratory phase before the onset of non-invasive ventilation (PEEP = 0). The two videos on the right demonstrate how the progressive increase in PEEP enhances lung expansion and aeration throughout the entire respiratory cycle. Video S3: Patient with Primary Non-Function and hemodynamic instability following liver transplantation. Ventilatory pressures were gradually increased to limit hemodynamic instabilities and prevent the compromise of hepatic outflow. With the sequential increase of PEEP from 8 to 12, alveolar ventilation improved, and the P/F ratio increased from 100 to 160. Ultrasound scanning was conducted in the second thoracic quadrant (see Figure 2) using an anterior intercostal approach with a linear probe and transverse scanning. The video illustrates the gradual improvement in ventilation of the consolidated area alongside the increase in PEEP. Video S4: Hyperinflation area in a patient with ARDS (A-lines > 5 with minimal pleural sliding visible). (A) Transition from an area with reduced ventilation to a hyperinflated area (longitudinal scan). (B) Transverse scan of hyperinflated area. Video S5: Before proceeding with alveolar recruitment in a consolidated area, it is helpful to assess airway patency, as demonstrated by the presence of air bronchograms (5a). Fluid bronchograms (marked with * in the video indicate the presence of secretions obstructing the bronchi. In the case of fluid bronchograms, alveolar recruitment manoeuvres are ineffective and should be preceded by bronchial de-obstruction manoeuvres (e.g., fibre-optic bronchoscopy). (S5A) Using a right intercostal approach, an ultrasound scan was performed with a convex probe in the fourth thoracic quadrant (see Figure 2). The video reveals extensive lung consolidation accompanied by dynamic air bronchograms. (S5B) An ultrasound scan was conducted with a transesophageal probe (TELU) in the mid-esophageal tract of the left lung. The video depicts lung consolidation with fluid bronchograms (*) indicating bronchial obstruction. The fluid bronchograms were distinguished from pulmonary vessels using colour Doppler ultrasound, as no Doppler signal is detected in fluid bronchograms. Video S6: Failed respiratory weaning attempt in a patient with diaphragmatic dysfunction. The diaphragm (D) shows a poor thickening fraction (mean Tfdi = 7%) and limited mobility (cranio-caudal excursion of 0.7 cm measured in the subcostal window) during spontaneous breathing trials, along with concurrent activation of the accessory intercostal muscles (IM). These findings indicate insufficient ventilatory support and predict weaning failure. R = rib, * = effusion in the costophrenic recess. Video S7: Ultrasonographic evaluation of the diaphragm during a spontaneous breathing test using a linear probe in the mid-axillary line. (A) The diaphragm shows standard thickness (36 mm, Tfdi = 38%) and excursion (cranio-caudal excursion of 2.1 cm measured in the subcostal window), indicating successful ventilatory weaning. (B) Diaphragmatic atrophy with significantly reduced thickness (1 mm), diminished diaphragmatic thickening fraction (no change in thickness), and limited excursion (cranio-caudal excursion of 4 mm measured in the subcostal window) in a patient with myopathy and prolonged mechanical ventilation. This condition resulted in weaning failure, necessitating tracheostomy. Video S8: Ultrasound-Guided Repositioning of the ETT in a Patient with Accidental Pulmonary Exclusion using Bianchini et al. technique [40]. The cranial edge of the cuff (*) is advanced to the cricoid cartilage (C). T = tracheal rings (1–2 are fused). Video S9: Preoperative ultrasound evaluation of the stomach in a patient with intestinal obstruction. Ultrasound findings revealed that the stomach was filled with fluid material. A nasogastric tube (NGT) was placed, and complete ultrasound-guided gastric emptying was performed preoperatively to reduce the risk of aspiration. Video S10: TELU demonstrates three pathological patterns in sequence: A/B pattern. Normal A-lines combined with B-lines arising from pleural lines in the context of an alveolar-interstitial syndrome. B/C pattern. B-lines combined with initial sub-pleural consolidation can be seen in various clinical situations (e.g., atelectasis, pneumonia, pulmonary contusion, pulmonary infarction). Pattern C combined with pleural effusion. The absence of alveolar air abolishes A-lines and B-lines, producing a tissue-like echotexture associated with anechoic homogeneous fluid (e.g., pneumonia with parapneumonic effusion).

## Abbreviations

The following abbreviations are used in this manuscript:

AHA	American Heart Association
ARDS	Acute Respiratory Distress Syndrome
CT	Computed Tomography
ESLD	End-Stage Liver Disease
ETT	Endo-Tracheal Tube
FOCUS	Focused Echocardiography
InsP	Inspiratory Pressure
ICP	Intracranial Pressure
LUS	Lung Ultrasound
LUSq	Lung Ultrasound Score Quantitative
LUSsc	Lung Ultrasound Score
NGT	Naso Gastric Tube
NIV	Non-Invasive Ventilation
PEEP	Positive End Expiratory Pressure
PIP	Peak Inspiratory Pressure
POCUS	Point of Care Ultrasound
PSV	Pressure Support Ventilation
RS	Spontaneous Respiration
SBT	Spontaneous Breathing Test
TELU	Transesophageal Lung Ultrasound
TFdi	Diaphragmatic Thickening Fraction
TFic	Intercostal Thickening Fraction
VExUS	score Venous Excess Ultrasound Score
VILI	Ventilator-Induced Lung Injury
VLUP	Visual Lung Ultrasound Protocol
